# The Effect and Mechanism of Magnesium Valproate on Methamphetamine‐Addicted Rats

**DOI:** 10.1111/adb.70084

**Published:** 2025-09-22

**Authors:** Yuanrong Li, Lixin Wang, Youhui Sun, Xuyi Wang

**Affiliations:** ^1^ Department of Psychiatry, and National Clinical Research Center for Mental Disorders The Second Xiangya Hospital of Central South University Changsha Hunan China; ^2^ Department of Psychiatry Anhui No. 2 Provincial People's Hospital Hefei Anhui China; ^3^ Department of Psychiatry Jiangxi Provincial People's Hospital, The First Affiliated Hospital of Nanchang Medical College Nanchang Jiangxi China

**Keywords:** behavioural sensitisation, conditional position preference, DAT, GSK‐3β, magnesium valproate, methamphetamine addiction

## Abstract

Valproate may hold promise as a treatment for addiction. However, there are limited studies examining the effects of magnesium valproate (VPA‐Mg) on methamphetamine (MA) addiction, and the relevant mechanisms have not been thoroughly discussed. This study aims to explore the potential therapeutic effects of VPA‐Mg on MA addiction and to investigate its possible mechanisms. The effects of VPA‐Mg on MA addiction were investigated using conditioned place preference (CPP) and behavioural sensitisation models in rats. VPA‐Mg was administered during CPP formation and extinction phases to evaluate its effects on MA‐induced CPP formation and reinstatement. Behavioural sensitisation assessed the impact of VPA‐Mg during sensitisation induction and expression phases, with spontaneous activity and MA dosing optimised beforehand. Furthermore, western blotting was performed on brain regions including the prefrontal cortex (PFC), hippocampus (Hip), nucleus accumbens (NAc) and ventral tegmental area (VTA) to measure glycogen synthase kinase 3 beta (GSK‐3β) and dopamine transporter (DAT) protein levels. VPA‐Mg did not exhibit a significant impact on MA‐induced CPP formation. VPA‐Mg significantly reduced the establishment and expression of MA‐induced behavioural sensitisation (*p* < 0.01). Pre‐treatment with VPA‐Mg for 3 days before the expression period also inhibited sensitisation (*p* < 0.05). In addition, the ratio of p‐GSK‐3β to t‐GSK‐3β in the PFC, Hip and VTA of rats with behavioural sensitisation significantly decreased, and the expression of DAT decreased significantly (*p* < 0.01). VPA‐Mg can reverse the increase in GSK‐3β activity in the Hip and the decrease in DAT in the PFC and Hip caused by repeated MA use (*p* < 0.05). VPA‐Mg exhibits anti‐addictive effects on MA dependence and relapse prevention. GSK‐3β activation and DAT downregulation in addiction‐related brain regions (PFC, Hip, VTA) are closely linked to MA addiction, suggesting potential therapeutic targets. VPA‐Mg may exert its effects by modulating these pathways, particularly in the PFC and Hip.

## Graphical abstract

This study shows that magnesium valproate significantly inhibits methamphetamine‐induced behavioural sensitisation in rats, while reversing the increased GSK‐3β activity and decreased DAT expression in PFC, Hip and VTA, but has limited effect on conditioned place preference.

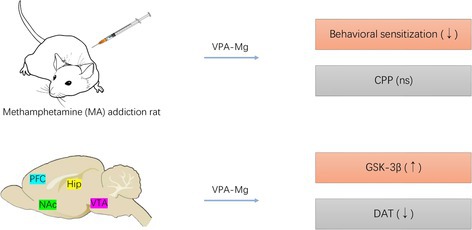

## 1 Introduction

Methamphetamine (MA), a prevalent synthetic drug and psychostimulant belonging to the amphetamine class, is a cationic lipophilic molecule that rapidly crosses the blood–brain barrier. It stimulates the central nervous system to release dopamine (DA), norepinephrine (NE) and 5‐serotonin (5‐HT), all of which are neurotransmitters closely associated with addiction [1, 2]. The brain is motivated to recognise and pursue rewards through the transfer of DA, glutamate (Glu) and gamma‐aminobutyric acid (GABA) within the structures that comprise the reward circuitry [3]. MA has become a widely abused substance globally. According to the World Drug Report 2022, global seizures of MA surged fivefold between 2010 and 2020. The China Drug Situation Report 2022 also highlighted that by the end of 2022, there were 1.124 million individuals in China who abused drugs. Among them, 588 000, accounting for 52.3%, were MA users, indicating that MA remained the most commonly abused substance in the country [4].

In animal studies, both behavioural sensitisation and conditioned place preference (CPP) are considered valuable models for studying addiction, although they reflect distinct aspects of the condition. Behavioural sensitisation is viewed as an external manifestation of neuroadaptation, indicating the brain's increased sensitivity to drugs and associated cues due to adaptive changes [2]. This phenomenon is linked to compulsive drug‐seeking behaviour and the potential for relapse [4]. Conversely, CPP focuses on assessing the rewarding effects of a drug by measuring the preference for the environment in which the drug was previously paired with it and is also often used to study reward‐related learning memory.

Valproate (VPA), a medication approved by the US Food and Drug Administration (FDA) for the treatment of epilepsy, bipolar disorder and migraine [5], has a multifaceted mechanism. In recent years, the inhibitory effect of VPA on glycogen synthase kinase 3 beta (GSK‐3β) has garnered extensive attention. GSK‐3β modulates behaviours induced by drug abuse and may serve as a potential target for the treatment of stimulant addiction [6]. Repeated use of psychoactive substances activates GSK‐3β in addiction‐related brain regions [7, 8, 9]. GSK‐3β inhibitors or VPA have been shown to alleviate behaviours related to addiction [10], and GSK‐3β has been identified as playing a crucial role in neuronal synaptic plasticity [11, 12]. In a study on MA‐induced behavioural sensitisation, increased GSK‐3β activity was observed in the rat nucleus accumbens core (NAcC). Systemic administration of the GSK‐3β inhibitor lithium chloride or microinjection of SB 216763 into the NAcC reduced MA‐induced sensitisation [13].

The current status indicates that (1) although the effects of VPA on addictive drug‐induced behavioural sensitisation have been reported in several studies, there is a lack of research on the associated neurochemical mechanisms; (2) there are fewer studies examining the effects of VPA on MA addiction; and (3) while all drugs currently used in animal studies are sodium valproate, it remains uncertain whether magnesium valproate has a similar effect.

To address these issues, this study used two animal models—behavioural sensitisation and CPP—to assess the effects of VPA‐Mg on MA addiction, rather than the commonly used sodium valproate, and explore the underlying neurobiological mechanisms. Given the critical roles of GSK‐3β in drug addiction and neuroplasticity, along with DAT's direct involvement as a target of MA and its link to addictive behaviours, the study specifically focused on these proteins. Western blot analysis was employed to measure their expression in addiction‐related brain regions, aiming to elucidate the anti‐addiction effects and molecular mechanisms of VPA‐Mg. This research provides a scientific basis for developing novel treatment strategies.

## 2 Material and Methods

### 2.1 Animals

The subjects were male Sprague–Dawley (SD) rats, 6 weeks old, (weighting 230–260 g) provided by Hunan Slack Kingda Animal Co. The animals were housed in a room with alternating light and darkness (light from 7:00 to 21:00), maintained at a room temperature of 23 ± 2 °C and a humidity level of 50 ± 10%. Each cage housed no more than five animals. All experimental procedures were performed in accordance with the approval of the Ethics Committee for Animal Welfare of the Second Xiangya Hospital, Central South University.

### 2.2 Conditioned Place Preference

**FIGURE 1:**
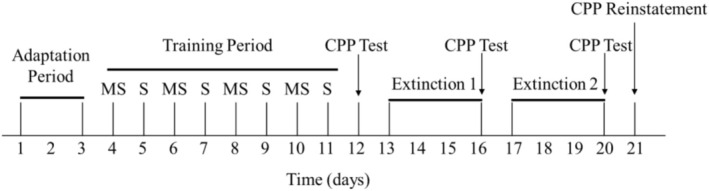
CPP experimental procedure.

The CPP apparatus comprises two different compartments (40 × 40 × 60 cm) and a central neutral area (40 × 6.5 × 40 cm). The procedure for the CPP experiment is depicted in Figure 1.

① Adaptation period (D1–D3): Rats explored the CPP device for 15 min daily, and their time in each compartment was recorded. On day 3, the preferred compartment was identified, and CPP values (disliked‐preferred compartment times) were calculated. Rats were randomly assigned to four groups: control, MA model, and two VPA‐Mg + MA groups (75 and 150 mg/kg), with eight rats in each group. No significant differences in CPP values were observed.
② Training period (D4–D11): The control group received saline gavage and injection; the MA model group received saline gavage and 2 mg/kg MA injection, while the VPA‐Mg (75, 150 mg/kg) + MA groups received the corresponding dose of VPA‐Mg gavage and 2 mg/kg MA injection. All injections were administered intraperitoneally. After injection, rats were placed in the disliked compartment (drug‐paired chamber) on odd‐numbered days and in the preferred (non‐drug‐paired chamber) on even‐numbered days, each for 45 min.
③ Formation period (D12): Rats were placed in the middle chamber for 15 min with access to all compartments, and time in each was recorded.
④ Extinction period: A conditioned extinction protocol was applied, with daily administration of the respective treatments and saline intraperitoneal injection. On odd‐numbered days, rats were placed in the drug‐paired chamber, and on even‐numbered days, they were placed in the non‐drug‐paired chamber, each for 45 min, until CPP was completely extinguished.
⑤ Ignition period: After CPP extinction, the control group received saline, and the other groups received 0.5 mg/kg MA. Rats were placed in the middle chamber, and CPP values were recalculated based on their compartment times.

### 2.3 Behavioural Sensitisation

The behavioural sensitisation device primarily consists of a camera, a spontaneous activity box (dimensions 45 cm × 45 cm × 40 cm), and a data acquisition system, which determines the rat's autonomous activity by recording and analysing the rat's activity trajectory in the testing area.

#### 2.3.1 Establishment of MA‐Induced Behavioural Sensitisation in Rats

The dosage of MA was initially evaluated during the formation and expression phases. During the formation period, SD rats received intraperitoneal injections of MA (0.5–4 mg/kg) or saline for six consecutive days. Following this, the rats were placed in a spontaneous activity box to measure their movement distance over 1 h. Subsequently, after an 8‐day transition period, the rats were injected with a low dose of MA (0.25–1 mg/kg), and their movement distance was measured again for 1 h.

#### 2.3.2 Effect of VPA‐Mg on Spontaneous Activity in Rats

The SD rats were acclimated to a spontaneous activity box for 2 days, 1 h daily. They were then randomly assigned to four groups based on their baseline locomotor activity (average of D1 and D2): the saline control group and three VPA‐Mg treatment groups receiving 75 mg/kg, 100 mg/kg and 125 mg/kg, respectively, with five rats in each group. There were no significant differences in baseline activity among the groups. The VPA‐Mg groups were administered VPA‐Mg via gavage every afternoon for three consecutive days, while the saline group received saline in the same manner. The following morning, the rats were placed back in the spontaneous activity box to measure their locomotor activity for 1 h.

#### 2.3.3 Effect of Concomitant VPA‐Mg During the Formation Period on the Development of MA‐Induced Behavioural Sensitisation in Rats

**FIGURE 2:**
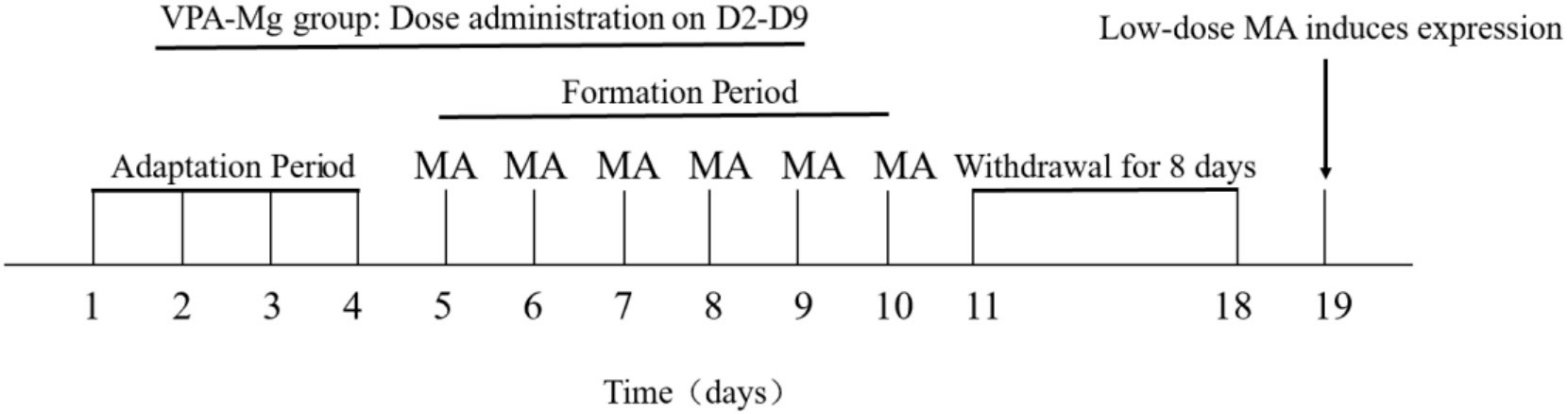
Experimental process of formation period with VPA‐Mg on MA‐induced behavioural sensitisation in rats.

① Adaptation period (D1–D4): SD rats were adapted to a spontaneous activity box for 1 h daily over 2 days. They were then randomly assigned to four groups—saline control, MA model and two VPA‐Mg + MA groups (75 mg/kg and 125 mg/kg)—based on baseline locomotor activity, with no significant differences between groups (*n* = 10–17 per group). Over the next 3 days, the control and MA model groups received saline, while the VPA‐Mg + MA groups received their respective doses via gavage each afternoon.
② Formation period (D5–D10): For 6 days, control rats received morning saline injections, while the MA model and VPA‐Mg + MA groups received 0.5 mg/kg MA and were tested for locomotor activity for 1 h. In the afternoons, the control and MA model groups received saline gavage, while VPA‐Mg + MA groups were gavaged with their respective doses.
③ Transition period (D11–D18): All rats were returned to their original cages after the formation period and were not administered any drugs for eight consecutive days.
④ Expression period (D19): Each group received 0.25 mg/kg MA and was tested for locomotor activity, with distance travelled measured over 1 h.
  The procedure is depicted in Figure 2.

#### 2.3.4 Effect of VPA‐Mg on the Expression of MA‐Induced Behavioural Sensitisation in Rats

① Adaptation period (D1–D2): The SD rats were adapted to a spontaneous activity box for 2 days, 1 h daily. They were then randomly assigned to four groups based on their baseline locomotor activity (the average of D1 and D2), which included a saline control group and a MA model group, with no significant differences in baseline activity observed among the groups.
② Formation period (D3–D8): For 6 days, the control rats received daily intraperitoneal saline injections, while the MA group rats were administered 0.5 mg/kg of MA and assessed for locomotor activity for 1 h. Based on the activity observed on day 8, the MA group was further divided into the MA model group and the VPA‐Mg (75 and 150 mg/kg) + MA groups.
③ Transition period (D9–D16): All rats were returned to their original cages without drug administration for eight consecutive days.
④ Expression period (D17): Each group received 0.25 mg/kg MA and was then placed in the experimental setup to measure distance travelled over 1 h.

**FIGURE 3:**
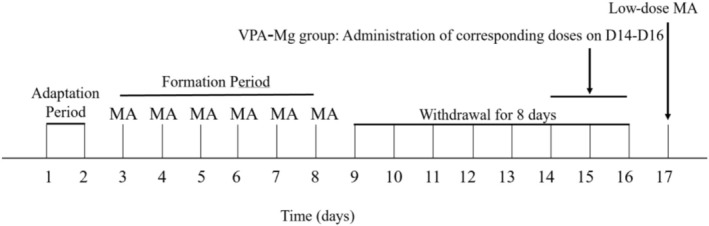
Experimental process of expression period with VPA‐Mg on MA‐induced behavioural sensitisation in rats.

The procedure is depicted in Figure 3.

### 2.4 Western Blotting

Brain tissues from rats, including the prefrontal cortex (PFC), hippocampus (Hip), nucleus accumbens (NAc) and ventral tegmental area (VTA), were rapidly collected and stored at −80 °C. The frozen tissue was chopped, mixed with pre‐cooled RIPA (Radio Immunoprecipitation Assay) lysis buffer (1:7 ratio), homogenised and lysed for 20 min. The lysate was centrifuged at 12 000 rpm for 10 min at 4 °C, and the supernatant was collected. Each group included 3 independent samples (*n* = 3) for protein extraction.

The protein concentration was determined using the BCA method. A standard protein gradient was prepared to generate a standard curve, and the protein concentration of the samples was calculated based on their absorbance values. After adding the loading buffer, samples were boiled, cooled and stored.

Ten percent SDS‐PAGE (sodium dodecyl sulfate polyacrylamide gel electrophoresis) separating gel and 5% stacking gel were prepared according to the molecular weights of the target proteins. The protein samples were loaded into the electrophoresis wells, and the voltage was initially set to 60 V for pre‐running. Once the blue dye front entered the separating gel, the voltage was increased to 90 V, and the running time was determined based on the molecular weight of the target proteins.

The target bands were cut from the gel, and NC (nitrocellulose) membranes were prepared. The transfer apparatus was assembled, and proteins were transferred to the NC membrane via electroblotting. The membrane was blocked with 5% BSA (bovine serum albumin) for 2 h, followed by washing with TBST (Tris‐buffered saline with Tween 20). The membrane was then incubated overnight at 4 °C with the primary antibodies, and the next day, it was washed again and incubated at room temperature with secondary antibodies for 2 h, followed by another wash with TBST.

ECL (enhanced chemiluminescence) substrate was added to the NC membrane, and the bands were scanned using a gel imaging system. The software was used for grayscale analysis.

### 2.5 Statistical Analysis

Statistical analysis and graphical representations were conducted using SPSS 26.0 and GraphPad Prism 9.0. All data are shown as mean ± SEM. The Shapiro–Wilk and Levene tests were used to assess normality and variance homogeneity, respectively. To compare means between three or more groups, a one‐way analysis of variance (ANOVA) was used, followed by LSD post hoc tests. The independent samples t‐test was performed to compare the two groups. The time‐course data for spontaneous activities and behavioural sensitisation were examined using two‐way repeated measures ANOVA using LSD for post hoc analysis.

## 3 Result

### 3.1 Conditioned Place Preference

#### 3.1.1 VPA‐Mg Does Not Affect MA‐Induced CPP Formation in Rats

**FIGURE 4:**
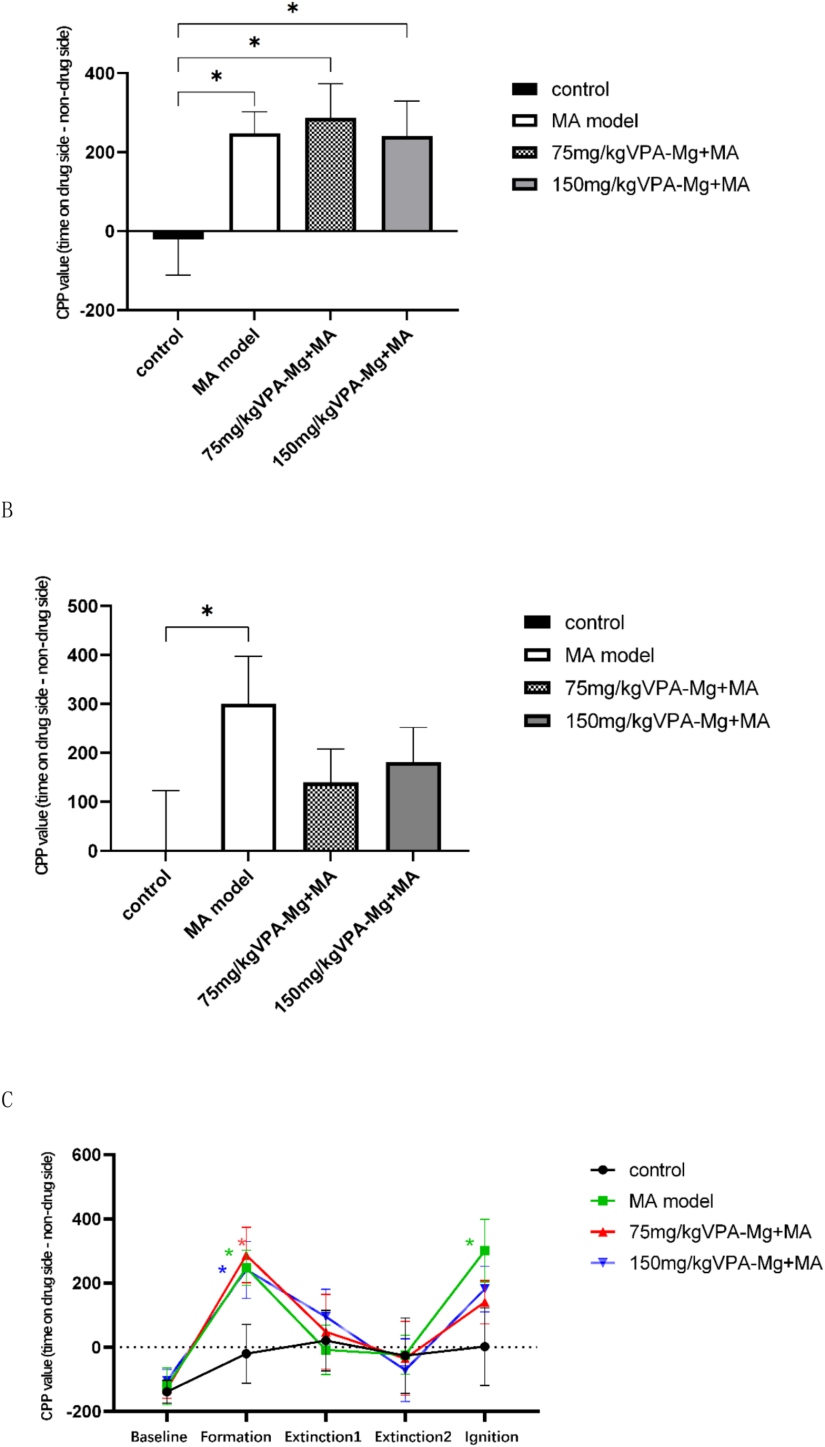
The impact of VPA‐Mg on MA‐induced CPP in rats. (A) The effect of magnesium valproate on the establishment of MA‐induced CPP in rats. (B) The effect of magnesium valproate on the reinstatement of MA‐induced CPP after extinction in rats. (C) Line graph showing changes in CPP values of rats in each group at different time points. *p < 0.05, compared with the control group at the same time point. Data are expressed as mean ± SEM, *n* = 8, analysed using one‐way ANOVA with LSD post hoc test. *p < 0.05.

Compared to the saline control group, CPP was significantly elevated in the MA model, 75 mg/kg VPA‐Mg + MA and 150 mg/kg VPA‐Mg + MA groups (*p < 0.05, Figure 4A). However, no significant differences in CPP values were observed between the VPA‐Mg + MA groups and the MA model group, indicating that neither 75 mg/kg nor 150 mg/kg of VPA‐Mg affected the establishment of MA‐induced CPP during the training period.

#### 3.1.2 The Impact of VPA‐Mg on the Reconstruction of MA‐Induced CPP in Rats Following Ablation Was Not Significant

After 8 days of conditioned withdrawal, CPP was fully extinguished with no significant differences between groups (Figure 4C). During the ignition period, saline was administered to the control group, while 0.5 mg/kg MA was given to the other groups to induce CPP reconstruction. As shown in Figure 4B, the CPP value in the MA model group was significantly higher than in the control group (*p < 0.05), confirming successful CPP re‐establishment. Although 75 mg/kg and 150 mg/kg VPA‐Mg reduced CPP values compared to the MA model group during extinction, the decrease was not statistically significant.

### 3.2 Behavioural Sensitisation

**FIGURE 5:**
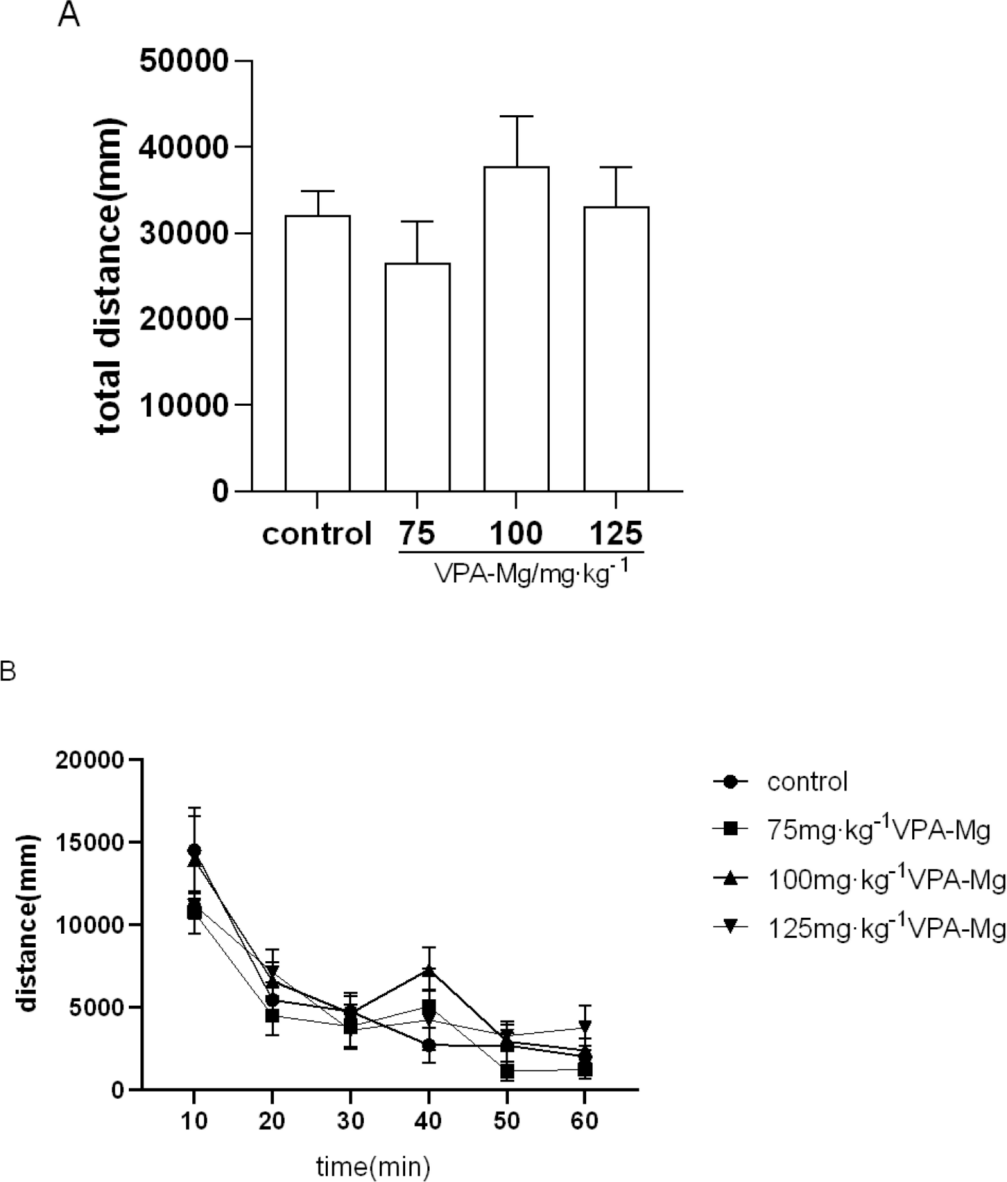
Effect of VPA‐Mg on spontaneous activity in rats. (A) One‐way ANOVA showed no significant difference in total locomotor distance between the saline control group and the 75, 100 and 125 mg/kg VPA‐Mg groups. (B) Two‐way repeated measures ANOVA indicated that there was no significant difference in locomotor distance between the saline control group and the 75, 100 and 125 mg/kg VPA‐Mg groups at the same time points.

First, our study showed that 0.5 mg/kg MA induced the establishment of behavioural sensitisation in rats. Moreover, 75, 100 and 125 mg/kg VPA‐Mg continuous gavage for 3 days did not affect the basal voluntary activity of rats (Figure 5).

This suggests that continuous intragastric administration of 75, 100 and 125 mg/kg VPA‐Mg for 3 days does not affect the baseline spontaneous activity of the rats. Data are presented as mean ± SEM, *n* = 5.

#### 3.2.1 Co‐Administration of VPA‐Mg Inhibits MA‐Induced Behavioural Sensitisation Formation in Rats

**FIGURE 6:**
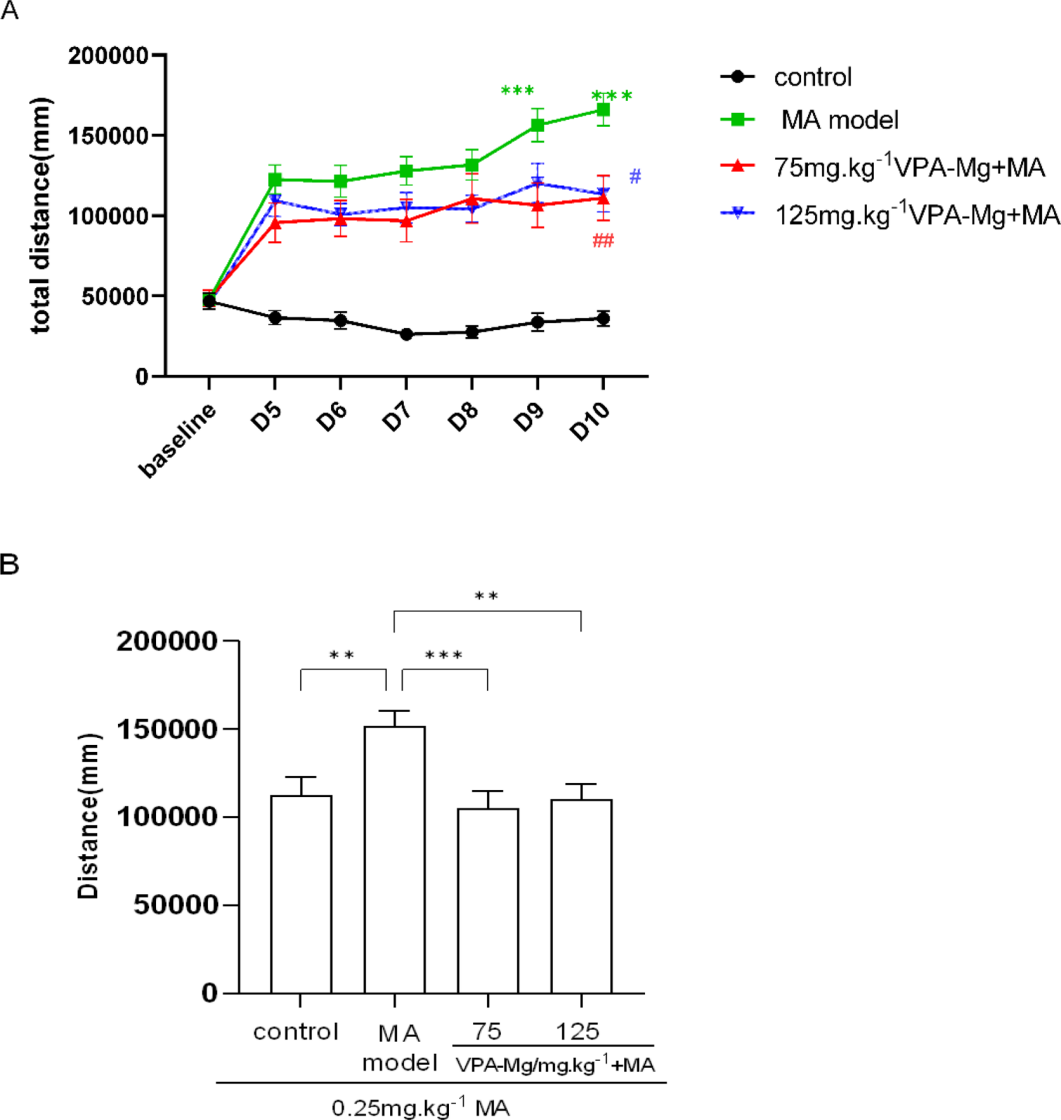
Effect of VPA‐Mg on the formation of MA‐induced behavioural sensitisation in rats. (A) Effect of VPA‐Mg on the formation period of MA‐induced behavioural sensitisation in rats: ***p < 0.001 compared to the same group on the first day of the formation period (D5), ##*p* < 0.01 and #*p* < 0.05 compared to the MA model group. Data are presented as mean ± SEM, *n* = 10–17, analysed using two‐way repeated measures ANOVA with LSD post hoc test. (B) Locomotor distance of rats induced by low‐dose MA after withdrawal: data are presented as mean ± SEM, *n* = 10–17, analysed using one‐way ANOVA with LSD post hoc test. ***p < 0.001, **p < 0.01.

Two‐factor repeated‐measures ANOVA showed that during the formative period, both the 75 mg/kg and 125 mg/kg VPA‐Mg + MA groups had significantly reduced locomotor activity compared to the MA model group (##*p* < 0.01, #*p* < 0.05; Figure 6A). On days 5 and 6, the MA model group exhibited a marked increase in movement compared to day 1 (***p < 0.001), while no significant changes were observed in the other groups. After 8 days of abstinence, 0.25 mg/kg MA re‐induction significantly increased locomotor activity in the MA model group compared to controls (**p < 0.01; Figure 6B), confirming successful behavioural sensitisation. Both VPA‐Mg treatment groups showed a significant reduction in movement distance compared to the MA model group (***p < 0.001, **p < 0.01).

#### 3.2.2 Pre‐Expression Administration of VPA‐Mg Inhibits MA‐Induced Behavioural Sensitisation in Rats

**FIGURE 7:**
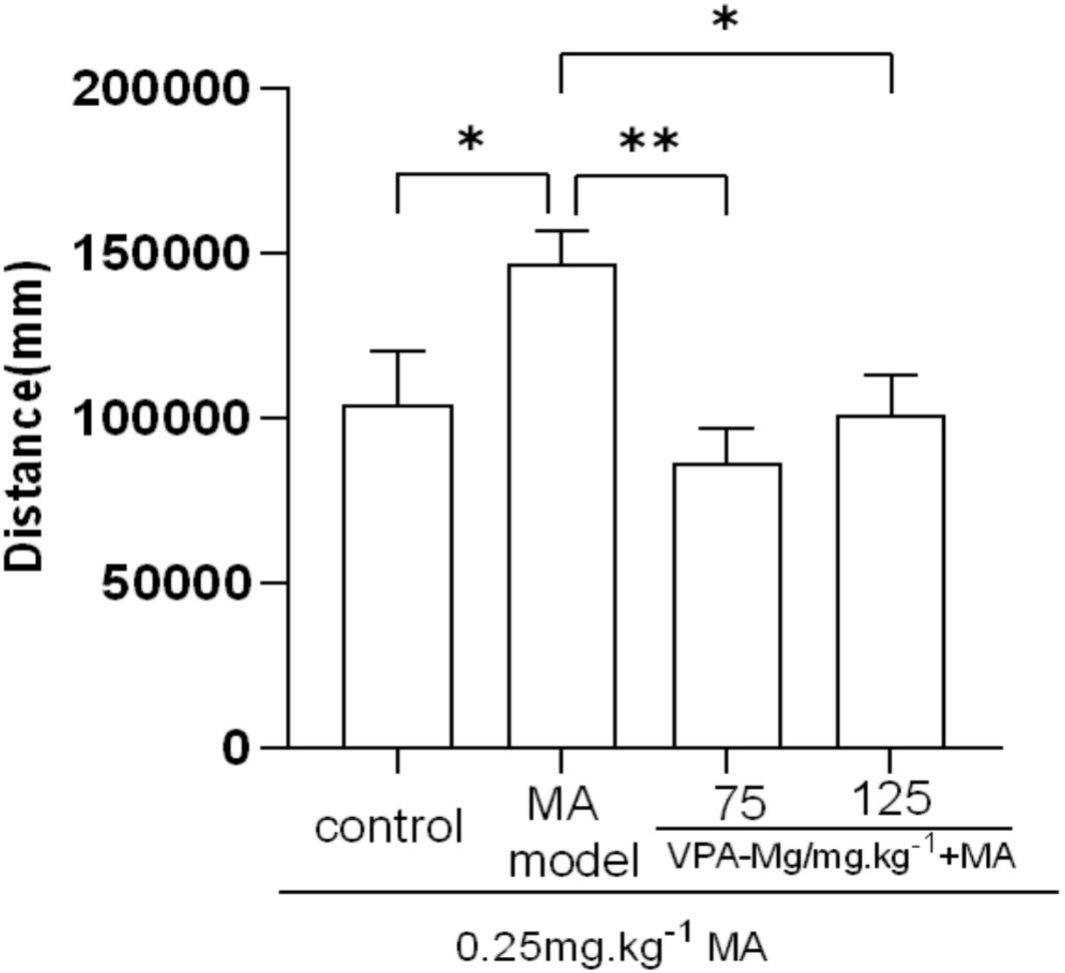
Effect of VPA‐Mg administration during the pre‐expression period on the expression of MA‐induced behavioural sensitisation in rats. Data are presented as mean ± SEM, *n* = 7–10, analysed using one‐way ANOVA with LSD post hoc test. **p < 0.01, *p < 0.05.

Expression was induced in SD rats by administering MA for 6 consecutive days, followed by a small dose of MA (0.25 mg/kg) after 8 days of withdrawal, which significantly increased locomotor distance in the MA model group compared to controls (*p < 0.05, Figure 7). Pre‐treatment with 75 mg/kg and 125 mg/kg VPA‐Mg for 3 days significantly reduced this increase (**p < 0.01, *p < 0.05), indicating that VPA‐Mg effectively inhibits MA‐induced behavioural sensitisation in rats.

### 3.3 Western Blotting

#### 3.3.1 Differences in the Expression of p‐GSK‐3β, t‐GSK‐3β and DAT Proteins in the PFC

**FIGURE 8:**
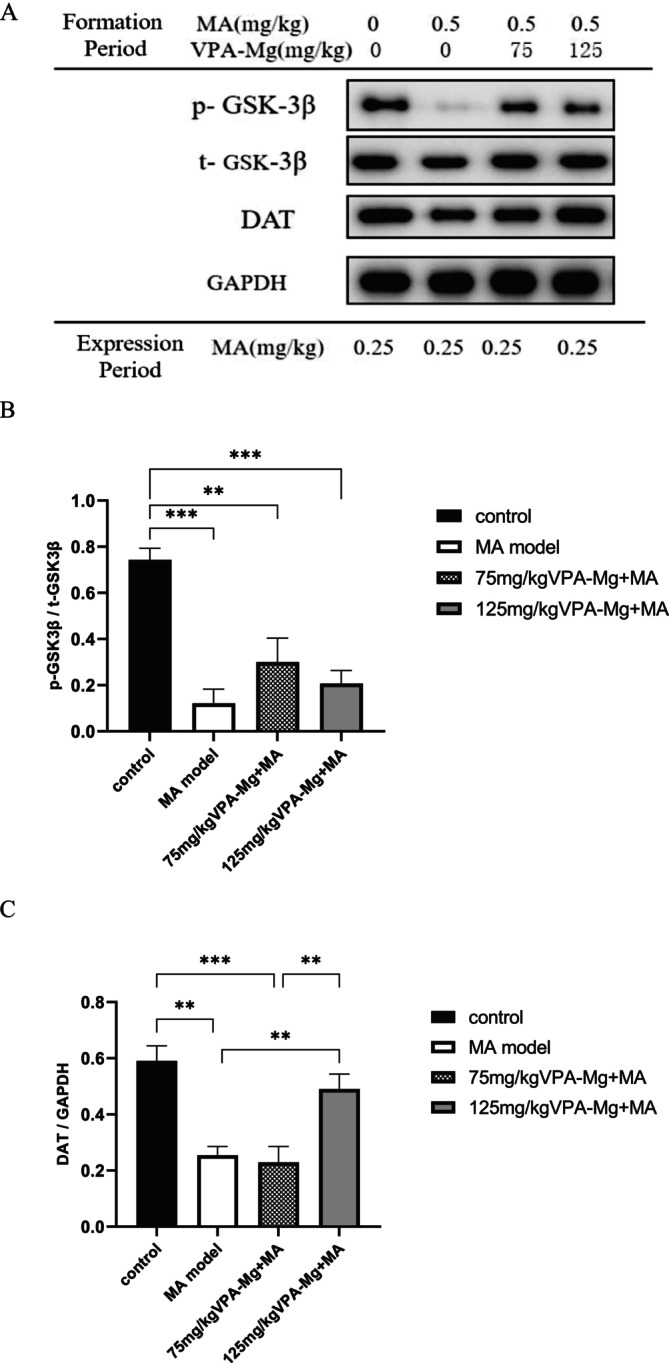
Expression levels of p‐GSK‐3β, t‐GSK‐3β and DAT proteins in the PFC of each group of rats. (A) Protein bands detected using western blot. (B) The ratio of phosphorylated GSK‐3β to total GSK‐3β protein. (C) Relative expression of DAT in the PFC. Data are presented as mean ± SEM, *n* = 3, analysed using one‐way ANOVA with LSD post hoc test. **p < 0.01, ***p < 0.001.

As shown in Figure 8A, p‐GSK‐3β, t‐GSK‐3β and DAT proteins were detected in the PFC across all groups. The MA model group showed a significantly reduced p‐GSK‐3β/t‐GSK‐3β ratio compared to controls (***p < 0.001, Figure 8B). Although the VPA‐Mg treatment (75 and 125 mg/kg) increased this ratio during the formation period, the change was not statistically significant (*p* > 0.05, Figure 8B). Regarding DAT expression, 75 mg/kg VPA‐Mg had no significant effect (*p* > 0.05, Figure 8C), while 125 mg/kg VPA‐Mg significantly increased DAT levels (**p < 0.01, Figure 8C).

#### 3.3.2 Differences in the Expression of p‐GSK‐3β, t‐GSK‐3β and DAT Proteins in the Hip

**FIGURE 9:**
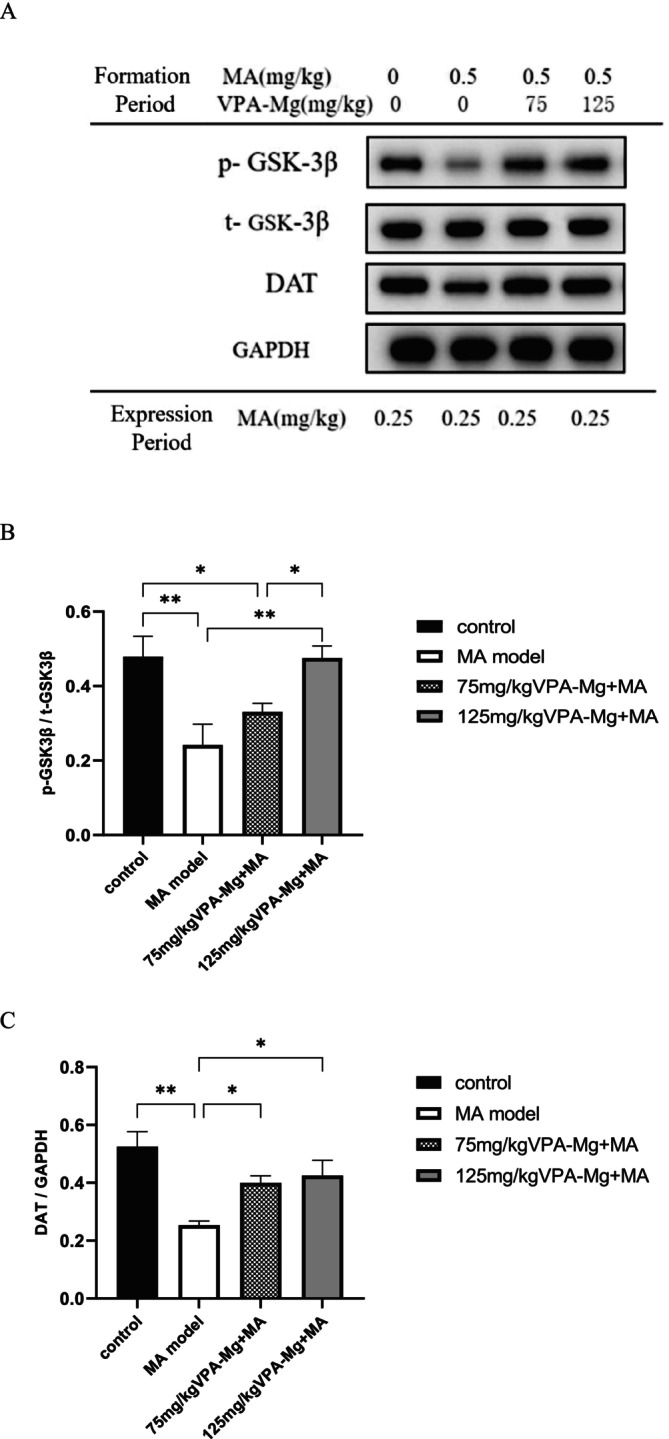
Expression levels of p‐GSK‐3β, t‐GSK‐3β and DAT proteins in the Hip of each group of rats. (A) Protein bands detected using western blot. (B) The ratio of phosphorylated GSK‐3β to total GSK‐3β protein. (C) Relative expression of DAT in the Hip. Data are presented as mean ± SEM, *n* = 3, analysed using one‐way ANOVA with LSD post hoc test. **p < 0.01, *p < 0.05.

As shown in Figure 9A, p‐GSK‐3β, t‐GSK‐3β and DAT proteins were expressed in the Hip across all groups. In the MA model group, the p‐GSK‐3β/t‐GSK‐3β ratio and DAT protein levels were significantly reduced compared to controls (**p < 0.01, Figure 9B,C). Co‐administration of 125 mg/kg VPA‐Mg during the formation period significantly increased the p‐GSK‐3β/t‐GSK‐3β ratio (**p < 0.01, Figure 9B), while 75 mg/kg had no significant effect (*p* > 0.05, Figure 9B). Both VPA‐Mg doses, however, significantly boosted DAT expression in the hippocampus (*p < 0.05, Figure 9C).

#### 3.3.3 Differences in the Expression of p‐GSK‐3β, t‐GSK‐3β and DAT Proteins in the NAc

**FIGURE 10:**
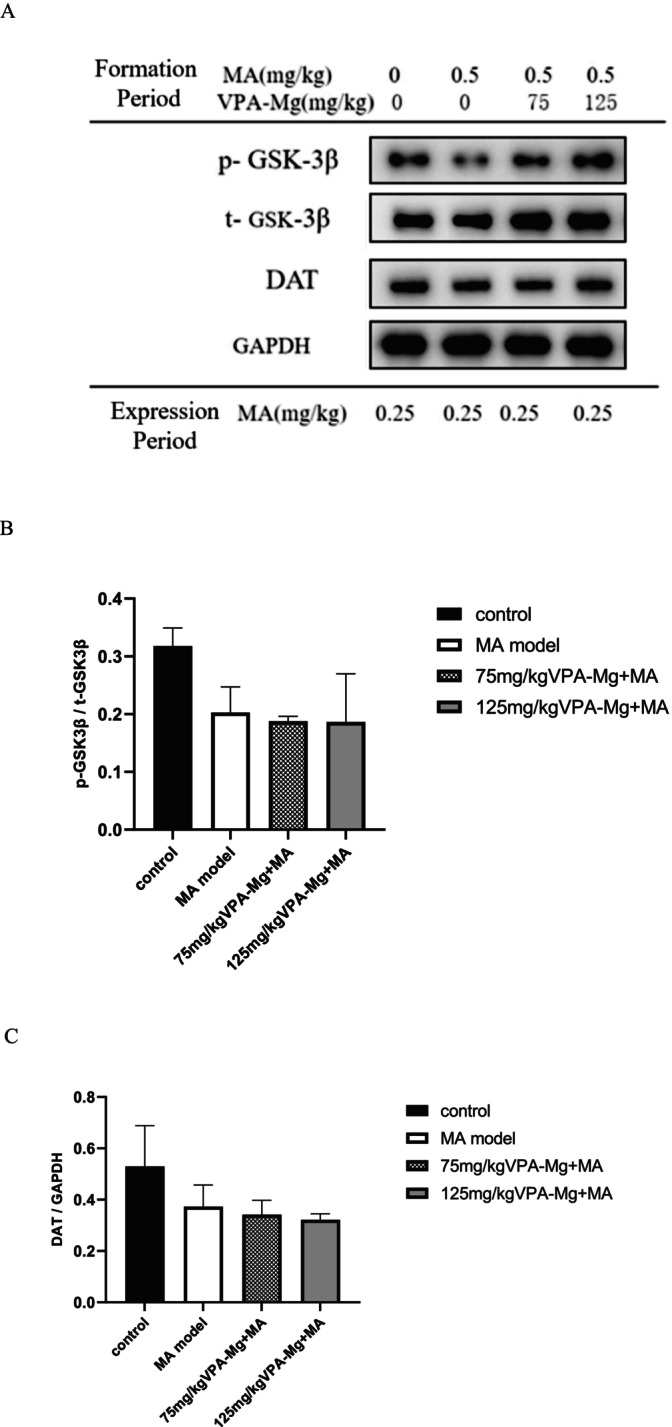
Expression levels of p‐GSK‐3β, t‐GSK‐3β and DAT proteins in the NAc of each group of rats. (A) Protein bands detected by western blot. (B) The ratio of phosphorylated GSK‐3β to total GSK‐3β protein. (C) Relative expression of DAT in the NAc. Data are presented as mean ± SEM, *n* = 3, analysed using one‐way ANOVA with LSD post hoc test.

As shown in Figure 10A, p‐GSK‐3β, t‐GSK‐3β and DAT proteins were expressed in the NAc of all groups. The MA model group exhibited a trend of reduced p‐GSK‐3β/t‐GSK‐3β ratios and DAT expression compared to the control group, though these changes were not statistically significant (Figure 10B,C). Similarly, administration of 75 and 125 mg/kg VPA‐Mg during the formation phase did not significantly affect these measures compared to the MA model group (Figure 10B,C).

#### 3.3.4 Differences in the Expression of p‐GSK‐3β, t‐GSK‐3β and DAT Proteins in the VTA

**FIGURE 11:**
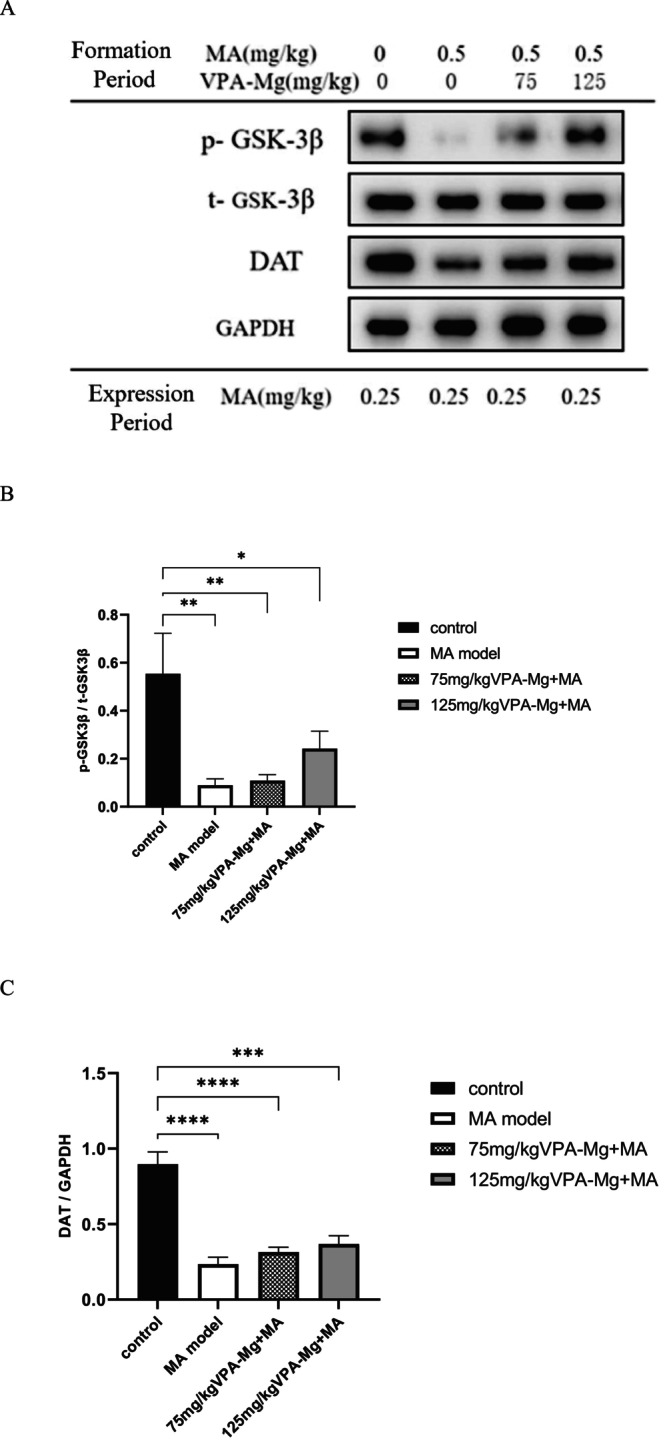
Expression levels of p‐GSK‐3β, t‐GSK‐3β and DAT proteins in the VTA of each group of rats. (A) Protein bands detected by western blot. (B) The ratio of phosphorylated GSK‐3β to total GSK‐3β protein. (C) Relative expression of DAT in the VTA. Data are presented as mean ± SEM, *n* = 3, analysed using one‐way ANOVA with LSD post hoc test. ****p < 0.0001, ***p < 0.001, **p < 0.01, *p < 0.05.

As shown in Figure 11A, p‐GSK‐3β, t‐GSK‐3β and DAT proteins were expressed in the VTA of all groups. In the MA model group, the p‐GSK‐3β/t‐GSK‐3β ratio and DAT protein levels were significantly reduced compared to the control group (**p < 0.01 and ****p < 0.0001, Figure 11B,C). Co‐administration of 75 and 125 mg/kg VPA‐Mg during the formation phase led to increases in these markers, but the changes were not statistically significant (Figure 11B,C).

## 4 Discussion

This study examined the effects of VPA‐Mg on behaviours related to MA addiction and explored potential mechanisms using the CPP and behavioural sensitisation models. Key findings include the following: (1) Administration of VPA‐Mg did not affect the formation of MA‐induced CPP and only slightly inhibited its re‐establishment at low doses during CPP recession. (2) VPA‐Mg significantly reduced the development of MA‐induced behavioural sensitisation during the formation stage and prevented sensitisation from low doses of MA when administered 3 days before expression. (3) In behavioural sensitisation, the p‐GSK‐3β/t‐GSK‐3β ratio and DAT expression were significantly reduced in the PFC, Hip and VTA, with similar trends in the NAc that were not statistically significant. VPA‐Mg reversed the increase in GSK‐3β activity in the Hip and the decrease in DAT expression in the PFC and Hip caused by repeated MA administration.

### 4.1 Effects of VPA‐Mg on MA‐Induced behavioural Sensitisation and CPP in Rats

Repeated and intermittent administration of MA to animals has been shown to increase psychomotor responses, such as autonomous activities, rotational behaviours and stereotyped locomotor patterns, indicating behavioural sensitisation. Investigators have often explored the neurobiological mechanisms of MA addiction through behavioural sensitisation [14]. This model, based on the incentive‐sensitisation theory by Robinson et al. [15, 16], posits that repeated drug use alters brain systems related to motivation, reward and decision‐making [17]. These alterations increase the drug's ‘incentive salience’ [18] and accompanying cues, resulting in compulsive drug‐seeking behaviour. Behavioural sensitisation is often used as evidence that the sensitisation of incentive motivational circuits takes place [19, 20]. In the present study, VPA‐Mg was found to inhibit MA‐induced behavioural sensitisation in rats, suggesting that it may prevent the sensitisation of brain circuits involved in incentive motivation.

Pathological motivation, known as incentive sensitisation, arises from the sensitisation of brain circuits driving motivational processes. However, associative learning influences the expression of neural sensitisation in specific environments [21], explaining why behavioural sensitisation typically only occurs in drug‐associated settings [22, 23]. This phenomenon mirrors how addicts relapse due to strong motivation triggered by drug‐related contexts [24, 25]. Behavioural sensitisation, therefore, helps explain cue‐induced cravings and the resumption of drug‐seeking behaviour.

Our study found that administering 75 or 125 mg/kg VPA‐Mg to rats for three consecutive days before the expression period significantly inhibited the expression of low‐dose MA‐induced behavioural sensitisation. Many studies have demonstrated that drug‐related cues and environments can induce a surge in DA and Glu release in addiction‐related brain regions [26, 27, 28, 29, 30], which triggers the compulsion to seek and consume drugs [31]. The intensity of craving triggered depends on both the reward association of the cue and the state of DA‐related brain systems in the individual, including factors such as stress and emotional arousal [32]. VPA‐Mg elevates brain GABA levels, inhibiting DA neuron activity in the VTA via GABA receptors, and dampening excitatory Glu signals in the NAc and VTA [33]. This suggests that VPA‐Mg may suppress sensitisation expression by reducing the cue‐induced surge of DA and Glu in the brain.

In contrast, 75 and 150 mg/kg VPA‐Mg did not influence CPP formation in rats induced by 2 mg/kg MA. The CPP assesses a drug's rewards by measuring preference for environments previously paired with the drug, suggesting that long‐term use of VPA‐Mg does not significantly counteract MA's rewarding effects. This may indicate that VPA‐Mg did not significantly interfere with the activation of reward circuits during the formative period, or that its regulatory mechanisms were not sufficient to counteract the strong excitatory effects produced by MA in the reward system. In addition, VPA‐Mg administered during the CPP recession phase showed a non‐significant trend toward inhibiting low‐dose MA‐induced CPP reinstatement, which could be attributed to the following possible reasons: (1) Current doses of VPA‐Mg may not effectively modify neuroplasticity changes in neural circuits involved in resuscitation memories. Higher doses and longer dosing cycles may be needed for more significant effects. (2) CPP reconstruction reflects the memory reactivation process associated with relapse behaviour, such as stress, reward, and conditioned reflexes. As a mood stabiliser, VPA‐Mg may be more effective at stabilising mood and regulating GABA balance than resuscitation dynamics driven by reward memory. (3) This result may also be limited by the small sample size. Thus, VPA‐Mg may influence MA addiction primarily by modulating the role of motivation, impulses, and cues in addiction, with less effect on the rewarding effects of the drug itself [34].

### 4.2 Neurobiological Research on the Effects of VPA‐Mg on MA Addiction

MA increases DA concentration in the synaptic cleft by inhibiting DAT, thereby activating the reward circuit [35]. Therefore, DAT is the main target of MA‐induced dependence and a potential target for addiction treatment [36]. Long‐term use of psychostimulants, such as MA and cocaine, decreases DAT protein levels [37], which is related to cognitive and motor disorders, as well as drug craving. Our study found that repeated MA exposure decreased the expression of DAT protein in addiction‐related brain areas (PFC, Hip, VTA, NAc), suggesting DA neuron damage. However, VPA‐Mg administration during MA exposure hindered the decrease of DAT protein in the PFC and Hip, indicating its neuroprotective effect.

Frey BN et al. have demonstrated that VPA can counteract MA‐induced oxidative stress and protect against neurotoxicity [38]. Drugs and related cue stimulation can induce a surge in DA in the brains of addicts [30]. DA in the PFC mediates behaviours like goal‐directed behaviour, reward learning, cognitive flexibility and executive functions [39, 40]. DA can modulate and integrate Glu synaptic transmission by activating D1 and D2R in the PFC [41]. Glu projections from the PFC to the NAc mediate compulsive drug seeking and relapse. When the DAT uptake effect in the PFC is weakened, more DA acts on D1 and D2R, which may damage the signalling between the PFC and NAc and lead to the failure of the PFC to control drug‐seeking behaviour. In conclusion, VPA‐Mg may reduce MA addiction by inhibiting the decline of DAT protein levels in the PFC.

GSK‐3 is a multifunctional serine/threonine kinase, originally identified as a regulator of glycogen metabolism and highly expressed in the mammalian brain [42], comprising two isoforms, GSK‐3α and GSK‐3β. GSK‐3β activity is tightly regulated by phosphorylation at the Ser9 site [11], which results in the inactivation of GSK‐3β. Conversely, dephosphorylation activates kinases that can phosphorylate GSK‐3β substrates, including transcription factors, structural proteins and signalling molecules. These substrates are critical for numerous cellular processes, such as the regulation of gene expression and synaptic plasticity [11, 12]. GSK‐3β, a key player in DA and Glu signalling, is a potential target for addiction therapy [4, 6]. In our experiment, western blot analysis indicated a significant decrease in the p‐GSK‐3β/t‐GSK‐3β ratio in the PFC, Hip and VTA, as well as a reduction in the NAc. These findings suggest that GSK‐3β was activated in the addiction‐related brain regions of sensitised rats, which aligns with the results of previous studies. DA projections from VTA to the NAc are the main neural circuit mediating the rewarding effects of MA, and some studies have also demonstrated that acute single administration of MA also activates GSK‐3β in the NAc, which may explain the lack of difference in GSK‐3β activity in the NAc of rats re‐exposed to MA versus those exposed to it for the first time in the present experiment.

Our experiment found that administering VPA‐Mg during the sensitisation formation period can reverse the MA‐induced increase in GSK‐3β activity in rat Hip and showed a downward trend in the PFC and VTA. Yan et al. also found that repeated MA use overactivates GSK‐3β in the Hip, leading to synaptic plasticity changes and impairments in memory‐related behaviours. Conversely, the GSK‐3β inhibitor reversed these deficits [43]. Glu projections from the Hip to the NAc regulate NAc neurotransmission, mediating drug reinforcement and cue‐induced memory retrieval [44, 45, 46]. Disrupting GSK‐3β signalling in the Hip may impair this regulation, inhibiting drug‐related memory retrieval and affecting addiction. Xing et al. also demonstrated that VPA microinjected into rats' NAc reduced acute MA‐induced hyperactivity and GSK‐3β activation [47]. However, our study did not observe significant effects of VPA on NAc GSK‐3β activity during sensitisation, likely due to differences in administration methods and protein assessment timing.

### 4.3 Limitations and Prospects

1. In the study on the impact of VPA‐Mg on MA‐induced CPP in rats, valproate did not influence CPP formation but seemed to inhibit its reformation during regression, albeit non‐significantly. Future studies could increase sample sizes and examine the effects of VPA‐Mg on reward memory retrieval and reconsolidation by administering valproate post‐drug or cue exposure.
2. Only GSK‐3β and DAT protein expression were detected in this study. In the future, protein expression can be detected at different periods, like the end of the sensitisation formation period and the withdrawal period, and it can be combined with techniques such as immunofluorescence and real‐time fluorescence quantification (qPCR).
3. . Our study only explored the activity of GSK‐3β. Future research could focus on studying upstream and downstream molecules related to GSK‐3β, such as protein kinase B (AKT) and mechanistic target of rapamycin (mTOR), to further clarify the role of GSK‐3β signalling in addiction. In addition, this study only examined DAT in the dopamine system, and further research could explore D1R, D2R, and D3R.

## 5 Conclusion

In conclusion, we demonstrate that VPA‐Mg exerts a certain effect on anti‐MA addiction and suppression of drug and related cue‐induced relapse. The activation of GSK‐3β and downregulation of DAT in addiction‐related brain regions such as the PFC, Hip and VTA are closely associated with methamphetamine addiction and may serve as potential therapeutic targets. VPA‐Mg could exert its therapeutic effect on methamphetamine addiction by inhibiting the activation of GSK‐3β and the downregulation of DAT in these brain regions.

## Author Contributions

**Xuyi Wang:** Conceptualization, Methodology, Supervision, Funding acquisition. **Yuanrong Li:** Writing – Original Draft, Writing – Review and Editing, Submission, Investigation. **Lixin Wang:** Methodology, Investigation, Data curation, Formal analysis. **Youhui Sun:** Investigation, Data curation.

## Conflicts of Interest

None of the authors have a conflict of interest to disclose.

## Data Availability

The data that support the findings of this study are available on request from the corresponding author. The data are not publicly available due to privacy or ethical restrictions.
